# Social-Media-Based Mental Health Interventions: Meta-Analysis of Randomized Controlled Trials

**DOI:** 10.2196/67953

**Published:** 2025-08-14

**Authors:** Qiyang Zhang, Zixuan Huang, Yuan Sui, Fu-Hung Lin, Hongjie Guan, Li Li, Ke Wang, Amanda Neitzel

**Affiliations:** 1 Yong Loo Lin School of Medicine, National University of Singapore, 21 Lower Kent Ridge Road, Singapore, 119077, Singapore, 65 66012186; 2 School of Education, Johns Hopkins University, Baltimore, MD, United States; 3 Department of Educational Policy Studies, College of Education and Human Development, Georgia State University, , Atlanta, GA, United States

**Keywords:** social-media-based interventions, depression, anxiety, systematic review, meta-analysis, randomized controlled trials

## Abstract

**Background:**

Compared with other forms of online mental health interventions, programs delivered through social media apps may require less training and be more acceptable and accessible to various populations. During and after the pandemic, both the number of social media users and the prevalence of social-media-based mental health interventions increased significantly. However, to the best of the authors’ knowledge, no meta-analysis so far has focused on rigorous social-media-based mental health interventions for general populations.

**Objective:**

This preregistered meta-analysis synthesized findings from rigorously designed randomized controlled trials (RCTs) (ie, decent sample size, low attrition, and comparable baseline conditions) to understand whether social-media-based mental health RCTs work as expected in reducing mental health issues.

**Methods:**

We searched for articles through database queries, hand searching, and forward and backward citation tracking, which yielded 11,658 studies. We only included social-media-based RCTs with a decent sample size (*n*≥30 for each experimental condition at baseline assessment), low differential attrition between treatments and controls (<15%), equivalent baseline conditions (differences between conditions <0.25 SDs), published after 2005, and delivered by nonresearchers. These RCTs must aim at reducing mental health issues, such as depression, anxiety, and stress. We excluded one-item outcome measures.

**Results:**

After double-blinded screening, 17 eligible studies (total sample sizes=5624) were included in this meta-analysis. Meta-regression results showed that, on average, these social-media-based interventions were effective (effect size [ES]=0.32, *P*<.001, N_ES_=61, 95% CI 0.24-0.45, *I*²=88.10, τ^2^=0.13) for the general population (range of mean age: 15.27~59.65). In other words, social-media-based interventions were effective at reducing anxiety (ES=0.33, *P*=.04, *n=*27), depression (ES=0.31, *P*<.001, *n*=31), and stress (ES=0.69, *P*=.02, *n*=12). Moderator analysis showed that social-media-based interventions are more effective when the participants are more than 70% female, when the programs are human-guided, social-oriented, and when control groups are care as usual. Furthermore, we conducted a risk of bias analysis, publication bias analysis, and sensitivity analysis, which show low risks of bias and robust findings. The biggest limitation of this review is the small sample size of 17 included studies, which restricts the power of our models.

**Conclusions:**

While technology can be a double-edged sword, this meta-analysis highlighted social media’s benefits and future potential in the treatment of mental health symptoms.

## Introduction

### Overview

More than 1 in 8 adults and adolescents worldwide live with a mental disorder, with anxiety and depression being the most commonly reported mental health symptoms [1]. Yet, a significant lack of access to mental health services has left more than 70% of those with mental disorders untreated [2,3]. The limited access to mental health services stems from widespread stigma surrounding mental health conditions, a lack of certified counselors and therapists [2,3], and a high employee turnover rate exacerbated by the global pandemic [4,5]. This global mental health crisis not only harms people’s physical health [6], personal wellness [7], and interpersonal relationships [8], but can also result in economic losses amounting to US $1 trillion [9].

Online mental health interventions delivered through social-media-based programs can potentially alleviate the mental health crisis. Traditional in-person mental health services often face limitations in accessibility, affordability, and reach. In comparison, social-media-based interventions can be delivered at scale [10], with a low cost [11], and require no extensive training [12]. These features facilitate people’s access to mental health resources and alleviate the burdens of health care workers. Furthermore, social-media-based interventions can also offer real-time peer support, access to resources (ie, psychoeducation), and therapeutic interactions in a familiar and engaging environment, creating a safe and supportive environment, especially for individuals who may feel stigmatized or isolated.

The growing popularity of social media in recent years presents an opportunity to critically evaluate its potential in promoting mental well-being [13]. However, while plenty of meta-analyses on social media interventions have focused on physical health-related outcomes such as sexual health and substance use [14], weight-related behavior [15], obesity [16], diet and exercise [17], there is a severe lack of research focusing on psychological health outcomes [18]. The pressing need to explore cost-effective interventions for mental health problems, especially with the current shortage of counselors [19] and the growing national crisis in mental health [20], makes our meta-analysis timely and necessary. In light of the common criticism of “garbage in, garbage out” on review work [21], this review only includes rigorously designed randomized controlled trials (RCTs) to provide high-quality evidence on what works. The results of this meta-analysis will give us an overview of the ways we can use social media to benefit our mental health.

### Past Meta-Analyses

Prior meta-analyses have examined extensively online [22,23], digital [24-27], eHealth [28], computer therapy [29], or internet-based [30-32] mental health interventions. However, up till the time of writing this paper, there are only two meta-analyses of social-media-based mental health interventions for patients with cancer [33,34], one scoping review probing into social-media-based mental health interventions for children [35], and a systematic review focusing on social networking sites in mental health interventions for young people [36]. To the best of the authors’ knowledge, no meta-analysis so far has focused on rigorously designed social-media-based mental health interventions for general populations. Therefore, we are motivated to conduct this meta-analysis.

### Potential Moderators in Interventions

Since there is no meta-analysis of social-media-based mental health interventions as a reference, we selected meaningful moderators based on past online and digital mental health interventions, a broader category that includes social-media-based interventions. We included the following 7 moderators.

1. Recruitment type: Recruitment type can be categorized into clinical or nonclinical, with the clinical approach selecting participants with health issues from health care facilities. The conflicting conclusions drawn from prior meta-analyses [31,37] necessitate a re-examination of whether intervention effects differ between clinical and nonclinical populations.
2. Age: Age is a critical proxy for health status, lifestyle, social media use, and internet efficacy. People of different age levels, for example, students versus older adults, may exhibit unique demographic and behavioral characteristics, which may lead to differential treatment effects [32,38].
3. Control group type: With a lack of consensus on the categorization of control group types, prior studies have conflicting conclusions on whether using active control groups (eg, placebos, diaries, behavioral recommendations) would yield different effect sizes (ESs) than using nonactive control groups (eg, waitlists, psychoeducational materials with no behavioral instructions, and passive controls) [31,39,40].
4. Intervention delivery personnel: Prior research also has mixed opinions on whether human guidance from personnel such as therapists, coaches, or research assistants would affect online intervention outcomes [31,41,42].
5. Intervention duration: Some systematic reviews fully or partially claimed that longer program duration increases intervention effects, with some studies fully or partially supporting this claim [39,41], while others disagree [31,42,43].
6. Program design (social-oriented or task-oriented programs): Task-oriented programs mainly assist with specific tasks, such as providing information or completing exercises. Social-oriented programs provide mainly social interaction, emotional support, or companionship. Therefore, social-oriented programs can provide more empathic conversations and warmth than task-oriented programs [44-46].
7. Sex: Sex can influence help-seeking behaviors [47] and preference for program types [48].

### This Meta-analysis

In this meta-analysis, we intend to answer two research questions.

- RQ1: What are the overall impacts of social-media-based RCTs on the alleviation of negative mental health outcomes (depression, anxiety, stress, negative affect, and psychological distress) for adolescents and adults when compared with care as usual (CAU) or waitlist?
  - H1: We hypothesize that social-media-based RCTs can effectively alleviate negative mental health outcomes.
- RQ2: To what extent do intervention outcomes differ according to methodological criteria, such as program duration and control group type (waitlisted vs CAU); program focus (social vs task-oriented); and intervention criteria, such as recruitment type (clinical vs nonclinical); intervention delivery (self-guided vs human guidance), sex, and age?
  - H2: We hypothesize that ESs will be larger in studies with a clinical population, younger age groups, more passive control groups, programs with human-guided support, social-oriented, more women, or longer intervention duration.

## Methods

### Registration

This meta-analysis was preregistered at Open Science Framework [49]. In practice, there were 3 deviations (population scope, intervention channel of focus, and outcome type) from our preregistered protocol. While the protocol focused on adults, we observed substantial variation in the age groups studied during the screening process. We also recognized that adolescents’ social media use patterns and platforms might differ from those of adults [50]. Thus, we decided to include studies involving all age ranges but conduct a moderator analysis using age groups.

The second change involves changing from including both internet-based and social-media-based interventions to including only social-media-based interventions. This decision was made to reduce heterogeneity in intervention delivery channels and allow for more in-depth analysis. Broader categories like mobile- or internet-based interventions include diverse formats such as digital platforms, chatbots, mHealth, and telehealth, which would have introduced significant variability. While numerous meta-analyses exist on general online or mobile mental health interventions, very few focus specifically on social media. To the best of our knowledge, the only existing meta-analyses on this topic target patients with cancer [33,34]. We identified this gap in the literature and adjusted our scope accordingly to contribute new and timely insights.

Last, we changed from including both positive and negative mental health outcomes to including only negative mental health outcomes. Our review found that study designs varied greatly depending on whether the intervention aimed to reduce negative mental health issues or enhance positive outcomes (eg, well-being, life satisfaction, and happiness), resulting in high heterogeneity. We also realized that enhancing positive mental health outcomes was not the same as reducing negative outcomes. For instance, the interventions that can effectively increase happiness are very different from interventions that can effectively reduce anxiety. To reduce heterogeneity, we chose to include only studies targeting negative mental health outcomes, such as depression, anxiety, stress, negative affect, and psychological distress.

### Search Strategy

To ensure comprehensive literature coverage, the first author (QZ) conducted a combined search strategy including database search, hand searching, and backward citation tracking. Using a predefined set of keywords on social media, intervention method, and mental health issues (Table S1 in Multimedia Appendix 1), the first author searched for literature in 7 databases, including the Education Resources Information Center, PsychINFO, Scopus, PsychArticles, Communication and Mass Media Complete, PubMed, and Proquest (Table S2 in Multimedia Appendix 1). Supplementary to the database search, targeted hand searching was performed through Paperfetcher [51] across selected reputable journals specific to the field (Table S3 in Multimedia Appendix 1). Finally, forward citation tracking and backward citation chasing were performed using CitationChaser on relevant systematic reviews and meta-analyses (Table S4 in Multimedia Appendix 1 [14,15,29,36,52-67]). We completed all literature searches and updates by April 2025, yielding a total of 11,658 studies, and subsequently imported them into Covidence for screening. Covidence was chosen for its functionality in facilitating full-text review and availability of software licenses through the authors’ affiliated institutions [68].

### Eligibility Criteria

We used the population, intervention, comparison, and outcome framework to help refine inclusion criteria. In this meta-analysis, we focused on general populations across different age groups and investigated mental health interventions delivered through social media platforms with either CAU or waitlist control groups. The outcomes of interest are negative mental health issues, such as depression, anxiety, stress, etc.

Eligible studies must meet the following twelve criteria to be included in this meta-analysis: (1) Studies must be RCTs. We excluded quasi-experimental study designs because we only want to include rigorous causal inference study designs. (2) Studies must have at least 30 participants per experimental condition at the baseline measurement. This is because extremely small samples can inflate ESs [69]. (3) Interventions must be largely delivered through social media platforms (eg, Facebook, Instagram, WhatsApp, and WeChat). We excluded social media abstinence interventions. (4) Based on the What Works Clearinghouse standards [70] for high-quality evidence, the difference between conditions at baseline on mental health measures must be less than 0.25 SDs. (5) Based on What Works Clearinghouse standards [70] for high-quality evidence, differential attrition between treatments and control groups must be less than 15%. (6) Intervention or instruction should be delivered by nonresearchers. This is because treatments delivered by researchers might be more challenging to sustain or replicate in the real world. For example, Guo et al [71] were excluded because the intervention was delivered by the authors. (7) Outcomes of interest measurements must include quantitative measures of negative mental health issues, such as depression, anxiety, stress, psychological distress, etc. The authors must either directly provide the ESs or offer enough statistics for us to compute Hedges g. For instance, Wang et al [72] were excluded because of insufficient data. (8) Full text must be available on the Internet and written in English. This is because we need to screen the full text to assess studies’ eligibility for inclusion, and our researchers need to double-code each study in English. (9) Articles must be published on or after January 1, 2005. This is because social media only started to be prevalently used after 2005 [73]. (10) Studies must be primary studies instead of secondary analyses. This prevents duplicated datasets from being included in this meta-analysis multiple times. (11) We excluded studies that use one-item measures because one-item measures are often unreliable [74]. (12) We excluded interventions that only have a single session because the extremely short program duration might inflate ESs.

### Screening and Coding Processes

Covidence was used for screening. All authors participated in the title and abstract screening as well as the full-text review. Each study was independently and double-blindly screened by at least 2 authors. Each eligible study was coded by 2 reviewers using Google Spreadsheets. The items extracted from each included paper can be found in Table S5 in Multimedia Appendix 1. We resolved any conflicts through weekly group discussions and reached a consensus with 100% agreement.

Following the call for open science, we shared all procedures, coding spreadsheets, and analysis code on GitHub [75] to ensure accessibility and future reproducibility.

### Analytical Plan

We used the R statistical software’s (R Foundation for Statistical Computing) metafor package for analysis [76]. This meta-analysis used weighted mean ESs and meta-analytic tests such as Q statistics. Weights were assigned to each study based on inverse variance [77] and were adjusted according to Hedges’ [78] recommendations. We used a random-effects model in meta-regression due to the presence of a range of ESs dependent on various factors [79]. For each primary study, we calculated standardized mean differences in Hedges’ g [80] through R’s metafor package’s function called escalc [76].

The moderator analysis included 7 sets of moderators. We grand-mean-centered all moderators and covariates to aid interpretation. Mean ESs were derived from the meta-regression model, which accounted for potential moderators and covariates. To assess publication bias, we used selection modeling instead of traditional methods (eg, funnel plot, Egger’s regression, fail-safe N) due to their limitations [81]. For instance, funnel plots are subject to meta-analysts’ interpretations, which are often misled by the plot shapes [81]. The fail-safe N technique is often criticized for the arbitrary choice of zero, which ignores heterogeneity in primary studies [82,83]. Selection modeling involved a weight function model implemented using the weightr package [84]. In adherence to the principles of open science, the complete description of the intervention characteristics is available together with the data and R code in GitHub [75].

As for risk of bias analysis, we applied the JBI Critical Appraisal Checklist for RCTs (Textbox 1 presents the 13 criteria). Since we applied stringent inclusion criteria, all the included studies already met the JBI Checklist’s criteria 1, 3, 7, 9, 10, 11, 12, and 13. Therefore, we only coded the criteria 2 (Was allocation to treatment groups concealed?), 4 (Were participants blind to treatment assignment?), 5 (Were those delivering treatment blind to treatment assignment?), 6 (Were outcomes assessors blind to treatment assignment?), and 8 (Was follow-up complete, and if not, were differences between groups in terms of their follow-up adequately described and analyzed?) to examine risk of bias. Following the double-blinded coding procedure, 2 independent reviewers conducted the quality assessment. A third reviewer was consulted to resolve the disagreement. In the online data repository [75], we listed the initials of the authors responsible for each study.

Textbox 1. **Risk of bias tool: JBI critical appraisal checklist for randomized controlled trials.**

Was true randomization used for the assignment of participants to treatment groups?

Was allocation to treatment groups concealed?

Were the treatment groups similar at the baseline?

Were participants blind to treatment assignment?

Were those delivering treatment blind to treatment assignment?

Were outcomes assessors blind to treatment assignment?

Were the treatment groups treated identically, other than the intervention of interest?

Was the follow-up complete, and if not, were differences between groups in terms of their follow-up adequately described and analyzed?

Were participants analyzed in the groups to which they were randomized?

Were outcomes measured in the same way for treatment groups?

Were outcomes measured in a reliable way?

Was an appropriate statistical analysis used?

Was the trial design appropriate, and were any deviations from the standard randomized controlled trial design (individual randomization, parallel groups) accounted for in the conduct and analysis of the trial?

### Seven Moderators

#### Recruitment Type: Clinical Versus Nonclinical

Interventions were coded as “non-clinical” if the participants were recruited from the general public or a specific subgroup of the general public (eg, health workers, college students) without a prescreening process for mental health symptoms. On the other hand, interventions were coded as “clinical” if participants were selected based on specific health conditions, such as people who passed a certain threshold for symptoms of depression, anxiety, or stress, or people who were referred based on their mental health clinical records.

#### Age: Adolescents Versus Early Adulthood Versus Middle Adulthood Versus Late Adulthood

We coded this variable based on the mean age of study participants. Study participants were coded to be “adolescents” if their average age was younger than 20 years, “early adulthood” if the average age ranges from 20 years to younger than 40 years, “middle adulthood” if the average age ranges from 40 years to younger than 60 years, and “late adulthood” if the average age is 60 years and older.

#### Control Group Type: Waitlist Versus Care as Usual

Control group type was coded as “waitlist” if studies used waitlist control groups. It was coded as “not waitlisted” if the control groups of the studies involved other types, such as treatment as usual or CAU.

#### Intervention Delivery: Self-Guided Versus Human-Assisted

Interventions were coded as “self-guided” if participants were given access to the social-media-based program but did not receive any human support or guidance. Conversely, interventions were coded as “guided by others” if they involved some kind of human help, ranging from email reminders to meetings with therapists, coaches, or research assistants who could give feedback, advice, or support.

#### Program Duration

We coded the duration from pretest to posttest and standardized the unit to weeks.

#### Sex: Seventy Percent Female

Seventy percent female was coded as 1 if at least 70% of the sample identified as female and 0 otherwise.

#### Social-Oriented or Task-Oriented Programs

Studies were coded based on social function as either “task-oriented” or “social-oriented.” The main aim of task-oriented studies was to help individuals accomplish particular tasks. In contrast, social-oriented studies focused on fostering social interaction, offering emotional support, or providing companionship, rather than targeting the completion of specific tasks.

## Results

### Search Process

**Figure 1.**
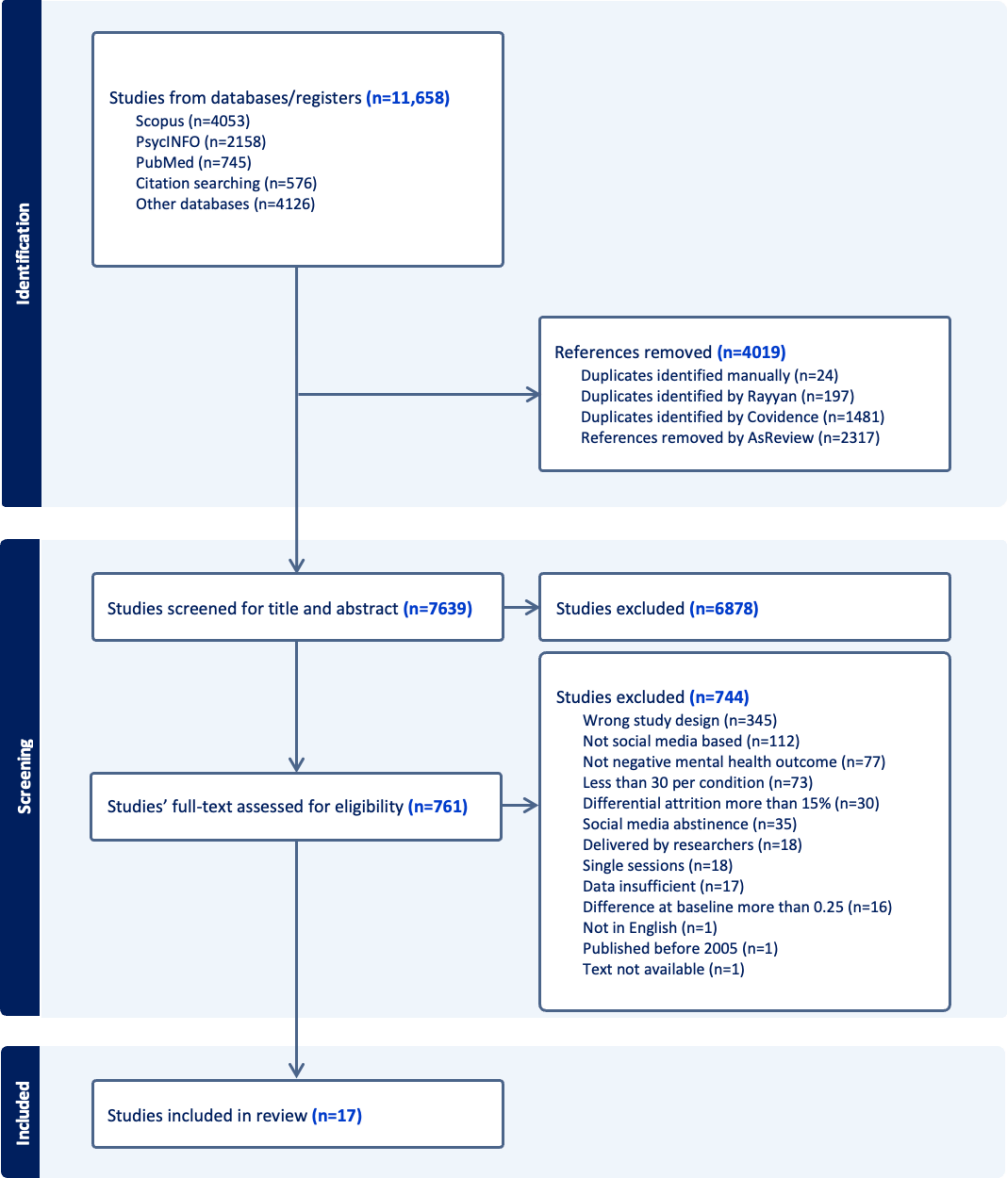
PRISMA (Preferred Reporting Items for Systematic reviews and Meta-Analyses) diagram.

The search strategy retrieved 11,658 published studies. Figure 1 presents the PRISMA (Preferred Reporting Items for Systematic reviews and Meta-Analyses) screening process (Checklist 1). A total of 4019 records were removed as duplicates. After deduplication, 7639 studies were screened by title and abstract, and 761 potentially eligible studies were reviewed in full text. During the full-text review stage, the top 5 reasons for exclusion included: wrong study design (*n*=345), not social media-based (*n*=112), absence of a negative mental health outcome (*n*=77), fewer than 30 participants per condition (*n*=73), and social media abstinence interventions (*n*=35). Finally, 17 studies were included in this meta-analysis. Table 1 presents the main characteristics of the included studies.

**Table 1.** Sample and treatment characteristics of the included 17 studies.

Study	Social media/website	Social or task	Self-guided	Age (years), mean (range)	Region	Sample size	Female, n (%)	Clinical	Control	Duration (Weeks)
Abedishargh et al [85]	WhatsApp	Task	0	34.4 (18‐50)	Iran	90	90 (100.00)	1	CAU^a^	24
Alvarez-Jimenez et al [86]	Horyzons	Social	0	20.91 (16‐27)	Australia	170	80 (47.06)	1	CAU	18
Chen et al [87]	WeChat	Social	0	59.65 (18 and older)	Mainland China	180	68 (37.78)	1	CAU	12
Duan et al [88]	WeChat	Social	0	32 (25‐44)	Mainland China	146	73 (50.00)	1	CAU	24
Garbett et al [89]	WhatsApp	Task	1	19.96 (15‐19)	Indonesia	1847	1847 (100.00)	0	Waitlist	1
Hatamleh et al [90]	WhatsApp	Task	0	31.5 (18‐45)	Jordan	128	128 (100.00)	1	CAU	8
Hemdi and Daley [91]	WhatsApp	Social	0	33.64 (23‐52)	Saudi Arabia	62	62 (100.00)	1	CAU	—^b^
Jane et al [92]	Facebook	Social	1	43 (21‐65)	Australia	91	—	0	CAU	24
Kang and Li [93]	WeChat	Task	0	65.2 (56‐74)	Mainland China	170	58 (34.12)	1	CAU	48
Lappalainen et al [94] (iACT)	WhatsApp	Social	0	15.27 (15‐16)	Finland	243	124 (51.03)	0	Waitlist	5
Lappalainen et al [94] (iACTface)	WhatsApp	Social	0	15.27 (15‐16)	Finland	243	124 (51.03)	0	Waitlist	5
Li et al [95] (dysregulated eating)	WhatsApp	Task	0	36 (18‐45)	Hong Kong	351	315 (89.74)	0	Waitlist	3
Li et al [95] (Insomnia)	WhatsApp	Task	0	42.13 (18‐45)	Hong Kong	333	265 (79.58)	0	Waitlist	3
Li et al [95] (Pain)	WhatsApp	Task	0	41.35 (18‐45)	Hong Kong	235	197 (83.83)	0	Waitlist	3
Lu et al [96]	WeChat	Task	0	59.7 (35‐82)	Mainland China	92	25 (27.17)	1	CAU	8
Mascarenhas et al [97]	Google Hangouts	Social	1	37 (18‐60)	United States	64	64 (100.00)	0	Waitlist	8
Pavarini et al [98]	Zoom and WhatsApp	Task	0	16.39 (16‐18)	United Kingdom	100	84 (84.00)	0	Waitlist	1
Prestin and Nabi [99] (comedy)	Youtube	Task	1	19.54 (16‐23)	United States	295	248 (84.07)	0	CAU	0.167
Prestin and Nabi [99] (Nature)	Youtube	Task	1	19.54 (16‐23)	United States	295	248 (84.07)	0	CAU	0.167
Prestin and Nabi [99] (underdog)	Youtube	Task	1	19.54 (16‐23)	United States	295	248 (84.07)	0	CAU	0.167
Xie et al [100]	WeChat	Social	0	41.98 (21‐62)	Mainland China	72	30 (41.67)	1	CAU	8.6
Yu et al [101]	Facebook	Social	0	20.53 (18‐23)	Taiwan	122	81 (66.39)	0	CAU	3

a CAU: care as usual.

b Not available.

### Descriptive Results

#### Study Characteristics

In total, 22 distinct intervention programs and 73 ESs were analyzed across the 17 studies (Table 2). Mental health outcomes included 31 (42.5%) ESs for depression, 27 (37.0%) for anxiety, 12 (16.4%) for stress, 2 (2.7%) for negative affect, and 1 (1.4%) for psychological distress.

**Table 2.** Descriptive statistics and distribution of the included studies’ features and outcomes.

Category and level	Overall, n (%)
Study level (total programs=22)	
Recruitment type	
Nonclinical	13 (59.1)
Clinical	9 (40.9)
Age (years)	
Adolescents	7 (31.8)
Early adulthood	7 (31.8)
Middle adulthood	7 (31.8)
Late adulthood	1 (4.5)
Sex	
Less than 70% female participants	10 (45.5)
More than 70% female participants	12 (54.5)
Program orientation	
Social	10 (45.5)
Task	12 (54.5)
Control group	
Care as usual	14 (63.6)
Waitlist	8 (36.4)
Delivery personnel	
Human guided	16 (72.7)
Self guided	6 (27.3)
Outcome level (total effect sizes=73)	
Outcomes	
Depression	31 (42.5)
Anxiety	27 (37.0)
Stress	12 (16.4)
Negative affect	2 (2.7)
Psychological distress	1 (1.4)

**Figure 2.**
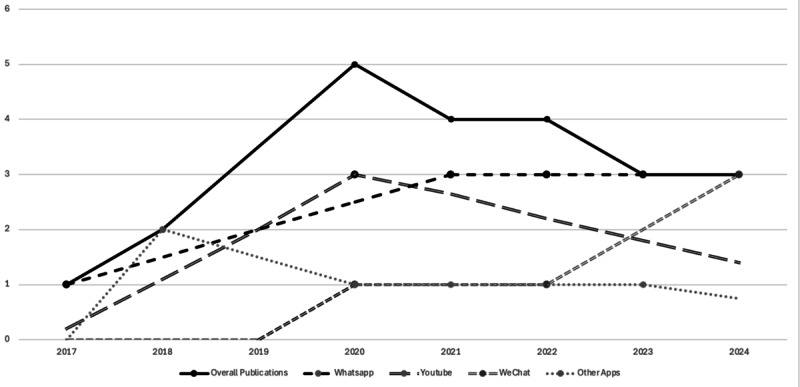
Distribution of included studies by year. Having completed our research by April 2025, the graph shows a limited number of 2025 studies. The included studies used mobile phones and examined different apps. The lines in the graph illustrate the number of publications related to WhatsApp, YouTube, WeChat, and other social media apps such as Facebook/Meta (n=2), Google Hangouts (n=1), and Zoom (n=1).

Figure 2 shows that the number of overall publications on social-media-based mental health RCTs meeting our criteria remained relatively low between 2017 and 2019. This reflects both the potentially early stage of high-quality research in this area. The period from 2020 to 2024 showed a notable increase in publications meeting our standards, peaking at 5 in 2020 and 4 in both 2021 and 2022. This surge strongly coincided with the COVID-19 pandemic, highlighting the interest in remote mental health intervention delivery via social media. However, the number of publications meeting our criteria decreased to 3 in both 2023 and 2024. This recent trend could be explained through a natural fluctuation in research output, a shift in research focus, or a lag in publication.

Among the included studies, the top 3 programs with the highest ESs are Hemdi and Daley [91], Hatamleh et al [90], and Abedishargh et al [85]. Hemdi and Daley’s [91] intervention consists of 4 self-guided WhatsApp-based sessions with therapist support for mothers of children with autism spectrum disorder. Hatamleh et al [90] used WhatsApp to create and deliver educational pamphlets and videos alongside their individualized interventions of a childbirth education program for primigravid Jordanian women. Abedishargh et al’s study [85] created WhatsApp groups for the 42 sessions of CBT-based therapeutic intervention. All 3 most effective programs were delivered through WhatsApp, highlighting this channel’s potential for future designs.

#### Treatment Characteristics

A total of 9 of the 17 studies involved clinical populations, while the remaining 8 studies focused on nonclinical groups. The most common recruitment method was through health care and professional networks (*n*=7, 41%), with participants recruited from hospitals, clinics, and specialized treatment centers. Recruitment via social media and online platforms (*n*=3, 18%), such as Facebook groups and email lists, was another commonly used method. Similarly, academic channels (*n*=3, 18%), including universities and secondary schools, served as other key recruitment sites. Flyers, posters, and direct communication (*n*=2, 12%) were used in a smaller number of studies, often through printed advertisements and in-person outreach. Last, 2 studies did not specify their recruitment methods (*n*=2, 12%). Full recruitment details for each study are available in the online data [75].

Cognitive Behavioral Therapy (CBT) refers to evidence-based psychotherapy delivered by trained clinicians to address acute symptoms of depression [102]. Only one intervention explicitly reported the use of CBT principles [85]. Regarding the orientation of the intervention content, 8 were task-oriented (eg, completing structured modules), while the other 9 were socially oriented (eg, enhancing social support or informal discussion spaces). Five studies used a waitlist control group, while 12 used nonwaitlist active controls, such as routine care or treatment as usual.

Delivery platforms showed variation across interventions. Among the 22 intervention programs, WhatsApp was the most commonly used (*n*=10), with one study combining it with Zoom and another integrating it with face-to-face sessions. Other platforms included WeChat (*n*=5), YouTube (*n*=3), Facebook (*n*=2), Google Hangouts (*n*=1), and Horyzons (*n*=1). In terms of guidance, the majority of interventions (*n*=15) were guided by facilitators, technicians, or professionals (ie, therapists and clinicians), while 6 were fully self-guided. One intervention was primarily guided by periodic reminders rather than active facilitation [101].

#### Sample Characteristics

Participants spanned a wide age range. A total of 7 programs targeted adolescents, 7 programs focused on individuals in early adulthood, and another 7 programs focused on middle adulthood, while 1 intervention focused on late adulthood.

The sex composition varied across studies, with 15 programs including samples in which females represented more than 50% of participants. Five programs only had female participants. The average percentage of female participants across studies was 70.02% (SD 25.08%), with the 25th percentile at 47.80% and the 75th percentile at 88.32%. We also found that studies that recruited 100% female participants were conducted in Iran, the Kingdom of Saudi Arabia, Jordan, Indonesia, and the US. In other words, all studies conducted in countries with predominantly Muslim populations recruited 100% female participants.

In terms of geographical distribution, 6 studies were conducted in Western, Educated, Industrialized, Rich, and Democratic (WEIRD) [103] countries, while 11 were from non-WEIRD countries. There was a diverse geographical distribution across countries, with most studies conducted in Greater China (Mainland China, Taiwan, and Hong Kong; *n*=8), followed by the United States (*n*=2), Australia (*n*=2), and other countries (*n*=5).

#### Meta-Analysis Results

As shown in Table 3, the overall mean ES for these 22 programs is 0.32 (*P*<.001) while holding all moderators fixed at their mean. The prediction interval shows that there is a 95% probability that a future observation will be contained within the prediction interval of −0.42 to 1.06. The CI shows that we are 95% certain that the true average effect lies between 0.18 and 0.46. Since the CI does not contain 0, we are more confident that social-media-based mental health interventions reduce negative mental health outcomes.

**Table 3.** Meta-analysis model results for models without and with moderators.

	Comparison groups	β	SE	*t* test (*df*)	*P* value
Null model					
Intercept	—^a^	0.32	0.07	4.49 (19.15)	<.001
Meta-regression					
Intercept	—	1.20	0.21	5.69 (4.93)	<.01^b^
Age: early adulthood	Age: adolescents	−0.39	0.21	−1.85 (4.25)	.13
Age: middle adulthood	Age: adolescents	−0.39	0.16	−2.40 (5.50)	.06
Age: late adulthood	Age: adolescents	0.72	0.30	2.39 (3.46)	.09
Program duration	—	−0.01	0.01	−1.31 (3.87)	.26
Program focus (task-oriented)	Program focus (social-oriented)	−0.76	0.20	−3.78 (3.24)	.03^c^
Waitlisted control	Care as usual	−0.49	0.11	−4.46 (3.11)	.02^c^
Self-guided	Human-guided	−0.72	0.19	−3.73 (3.71)	.02^c^
More than 70% female	Less than 70% female	1.40	0.30	4.62 (3.60)	.01^c^
Clinical population	Nonclinical population	0.34	0.20	1.65 (4.08)	.17

a Not applicable.

b *P* <.01.

c *P* <.05.

**Figure 3.**
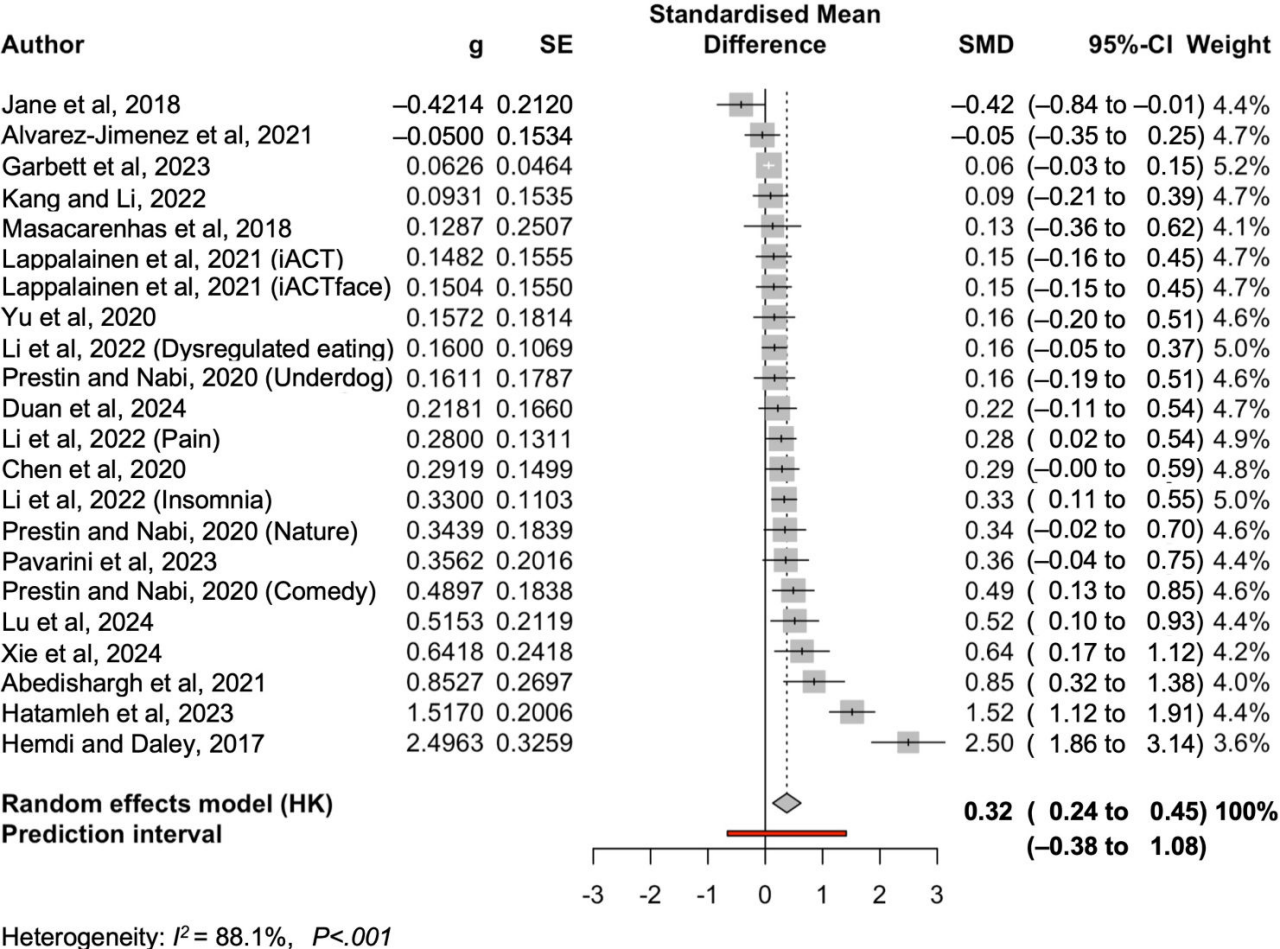
Forest plot [85-101].

The *I*² value is 88.1%, indicating that 88% of the total variability in effect estimates across studies is due to heterogeneity rather than chance, and the remaining 11.9% of variability is due to random chance. A closer investigation shows that partial *I*² is 28.87% (between studies) and 59.23% (within cluster heterogeneity). This means high heterogeneity, especially within the study. The *τ*^2^ is 0.13, which means a modest but meaningful degree of heterogeneity. Figure 3 presents the forest plot.

When it comes to outcome subgroups, on average, social-media-based mental health interventions are effective for depression (ES=0.31, *P*<.001, *n*=31), anxiety (ES=0.33, *P*=.04, *n=27*), and stress (ES=0.69, *P*=.02, *n*=12). Table 4 reports marginal means for each subgroup.

**Table 4.** Marginal means of the meta-regression model with moderators.

Moderators and groups	β	SE	*t* test (*df*)	*P* value
Clinical population				
0	1.04	0.23	4.60 (5.17)	<.01^a^
1	1.37	0.24	5.66 (3.19)	<.01^a^
Age (years)				
Adolescents	1.20	0.21	5.69 (4.93)	<.01^a^
Early adulthood	0.81	0.14	5.70 (3.53)	<.01^a^
Middle adulthood	0.81	0.15	5.52 (2.94)	.01^b^
Late adulthood	1.92	0.34	5.73 (3.28)	<.01^a^
Waitlisted control				
0	1.37	0.24	5.79 (5.46)	<.01^a^
1	0.88	0.18	5.01 (3.73)	<.01^a^
Program focus				
Social-oriented	1.20	0.21	5.69 (4.93)	<.01^a^
Task-oriented	0.44	0.11	4.07 (4.24)	.01^b^
Delivery personnel				
Human guided	1.35	0.24	5.55 (4.96)	<.01^a^
Self guided	0.63	0.13	4.69 (3.52)	.01^b^
More than 70% female				
0	0.41	0.13	3.28 (4.77)	.02^b^
1	1.81	0.33	5.54 (4.83)	<.01^a^

a *P* <.01.

b *P* <.05.

#### Age Groups: Adolescents Versus Early Adulthood Versus Middle Adulthood Versus Late Adulthood

The results showed no statistically significant moderation by age group. Compared with middle adulthood, social-media-based interventions might be more effective among adolescents; this comparison is approaching significance (*P*=.06). Late adulthood had the largest marginal mean ES (ES=1.92, *P*<.01), followed by adolescents (ES=1.20, *P*<.01).

#### Intervention Duration

The analysis showed that program duration did not significantly moderate the effect (*P*=.26). This suggests that longer or shorter social-media-based programs did not substantially influence outcomes.

#### Clinical Population: Clinical Versus Nonclinical

Whether participants were recruited from clinical settings did not significantly moderate the effect (*P*=.17).

#### Control Group Type: Waitlist Versus Other Types

Control group type significantly moderated the effect (*β*=−0.49, *P*=.02). This means that compared with active control and CAU (ES=1.37, *P*<.01), interventions where control groups are waitlist controls are less effective (ES=0.88, *P*<.01).

#### Delivery Personnel: Self-Guided Versus Human-Guided

 Delivery personnel significantly moderated the effect (*β*=−0.72, *P*=.02). This suggests that compared with programs with human guidance (ES=1.35, *P*<.01), self-guided programs were less effective (ES=0.63, *P*=.01).

#### Sex: Seventy Percent Female

Sex significantly moderated the effect (*β*=1.40, *P*=.01). Studies with majority-female participants (>70% female participants) showed significantly higher ESs. Marginal means further indicate a large difference when the sample is more than 70% female (ES=1.81, *P*=.003) and when the sample is less than 70% female (ES=0.41, *P*=.02).

#### Program Focus: Social Oriented Versus Task Oriented

Program focus significantly moderated the effect (*β*=−0.76, *P*=.03), indicating that social-oriented programs are more effective (ES=1.20, *P*=.002) than task-oriented programs (*β*=0.44, *P*=.01).

### Selection Bias Analysis

Applying the weight-function model, we found that the mean effect estimates were upwardly adjusted, implying that statistically nonsignificant effects were less likely to be reported than significant results (g is 0.98 when the cut-off point for *P* value is between .025 and .5 [ie, *P*<.01]; g is 0.02 when the cut-off point for *P* value is between .5 and 1 [ie, *P*=.28]). 

### Risk of Bias Analysis and Sensitivity Analysis

Table 5 presents the risk of bias analysis. Among the 17 included studies, 6 studies concealed allocation to treatment groups, 3 studies did not, and 8 studies were unclear. Four studies blinded participants to treatment assignment, 7 studies did not, and 6 studies were unclear. Only 2 studies blinded those delivering the treatment, 7 studies did not, and 8 studies were unclear. Four studies blinded outcomes assessors, 1 study did not, and 12 were unclear. Six studies completed follow-ups, while 11 did not clearly report this information. Overall, the risk of bias across the included studies can be considered low, as the mean appraisal score was relatively high (9.29 out of 13).

**Table 5.** Risk of bias (RoB) analysis. Since this review applies stringent inclusion criteria, RoB 1, 3, 7, 9, and 10‐13 are “Yes” for all cells. For clarity reasons, we omitted those columns to better present the RoB analysis.

Study	RoB 2	RoB 4	RoB 5	RoB 6	RoB 8	Overall appraisal score (total is 13)
Abedishargh et al [85]	Y^a^	Y	Y	Y	Y	13
Alvarez-Jimenez et al [86]	U^b^	Y	N^c^	Y	Y	11
Chen et al [87]	Y	N	N	U	U	9
Duan et al [88]	Y	N	N	U	U	9
Garbett et al [89]	U	U	U	U	U	8
Hatamleh et al [90]	U	N	N	U	Y	9
Hemdi and Daley [91]	Y	Y	U	Y	Y	12
Jane et al [92]	U	U	U	U	U	8
Kang and Li [93]	Y	U	U	U	U	8
Lappalainen et al [94]	U	U	U	U	U	8
Li et al [95]	N	N	N	N	U	8
Lu et al [96]	U	N	N	U	Y	9
Mascarenhas et al [97]	N	N	Y	U	Y	10
Pavarini et al [98]	Y	U	U	Y	U	10
Prestin and Nabi [99]	U	U	U	U	U	8
Xie et al [100]	N	N	N	Y	U	9
Yu et al [101]	U	Y	U	U	U	9

a Y: yes.

b U: unclear.

c N: no.

Since the 70% threshold for defining a majority-female sample is somewhat arbitrary, we conducted a sensitivity analysis using a 50% threshold instead. The result remained robust, indicating that studies with a majority-female participants were still more effective than those with a majority-male participants.

## Discussion

### Principal Findings

This meta-analysis found that rigorous social-media-based RCTs were effective in reducing mental health issues. In particular, social-media-based interventions are more effective when the participants are mainly female, when the programs are human-guided, social-oriented, and when control groups are CAU.

Figure 2 shows that the number of rigorously designed social-media-based mental health RCTs peaked from 2020 to 2024, reflecting the impact of the COVID-19 pandemic as well as the increasingly rigorous methods. After the pandemic, the number started dropping but remained higher than the prepandemic average. The trend in this research area highlights the need for review work to understand the overall picture of these interventions.

### Female-Only Recruitment in Muslim Countries

In descriptive results, interestingly, all studies conducted in Muslim countries (Iran, the Kingdom of Saudi Arabia, Jordan, and Indonesia) recruited 100% female participants. This might be attributed to gender roles and cultural expectations. The online channels might be more appealing to this population since Muslim women prefer to be seen by a female health care provider [104,105], which would not be a barrier in social-media-based programs. Research has shown that Muslim women are not only more vulnerable to mental health issues, but they are also more likely to experience mental health stigma and a lack of social support [106]. It is worth investigating whether the anonymity features on social media encourage more help-seeking behaviors among Muslim women and whether the researchers in the Middle East deliberately designed interventions targeting women.

### Social-Media-Based Interventions Were Effective at Reducing Mental Health Issues

The weighted-average ES showed that social-media-based interventions were effective at reducing depression, anxiety, stress, negative affects, and psychological distress. This reinforces previous meta-analyses conducted by Siew et al [33] and Wang et al [34]. Although they found a slightly larger ES (Siew et al [33]: 0.41 and Wang et al [34]: 1.1 and 1.37) compared with our 0.32, one reason could be their focus on patients with cancer only. Moreover, compared with general online and internet-based mental health interventions that found higher ESs, this social-media-based meta-analysis showed lower ESs. For instance, Christ et al [32] focused on CBT interventions only and reported ESs of 0.51 on depression and 0.44 on anxiety. Another example is Alrashdi et al [23] who found ESs of 0.41 on depression, 0.40 on stress, and 0.45 on anxiety. Since social-media-based mental health interventions are relatively new channels compared with digital and online interventions, the developing designs and implementation fidelity might affect the overall ESs.

### Interventions Are More Effective for Majority-Female Participants

Our moderator analysis showed that social-media-based interventions are more effective when the participants are more than 70% female. This is probably because women tend to obtain more social support from social media platforms [107] and engage more in an interactive and reflective community [47]. According to the tend-and-befriend theory, females give more social support than males when they experience stress, whereas men often respond to stress with the fight-or-flight mindset [108]. Since females give more social support, they might receive more social support due to the norm of reciprocity. Furthermore, females generally have higher rates of help-seeking and engagement with mental health resources than males [47].

In future studies, researchers could investigate more into how these social-media-based programs work differently for different sexes. It is possible that the type of social support provided through social media is more appealing and therapeutic for women [109]. However, this means that future interventions should seek answers for what works best for the majority of male participants. Readers should note that, among the 17 studies, only one study is of a majority-male sample (more than 70% male).

### Social-Oriented Programs Are More Effective Than Task-Oriented Programs

We found that programs that primarily provide social interaction are more effective than programs that provide exercises, information, and tasks. In mental health therapy, therapeutic relationships are important [45,46]. This moderator might also confound with the sex moderator since previous studies found that women prefer communicative channels while men prefer solution-focused or skills-training programs [47]. Since most programs have more than 50% female participants, the sex factor might have interacted with the program orientation.

### Programs With Human Guidance Are More Effective Than Self-Guided Programs

We found that programs guided by nurses, clinicians, coaches, or therapists are more effective than self-guided programs. This finding aligns with previous systematic reviews that guided programs, regardless of qualifications, are more effective than nonguided programs [110,111]. Support from others can enhance motivation, reduce attrition, and create therapeutic relationships for improved counseling effectiveness [112]. Human supporters can also hold users accountable to engage more in the designed programs by providing timely help, which eventually leads to more effectiveness of the program [113]. This shows that although social-media-based programs are less costly to scale up, incorporating certain human elements is essential in maintaining the programs’ value and effects. Another thing to note is that most social-oriented programs are human-guided (86%) rather than self-guided. It is possible that these 2 moderators’ effectiveness affects each other.

### Programs With Active and Care as Usual Controls Are More Effective Than Programs With Waitlist Control

This finding is unexpected and contradicts our hypothesis. While the conventional assumption is that participants assigned to the waitlist control will not engage in anything, this might not hold true for this topic. Most studies recruited people who voluntarily signed up through social media advertisements. Participants assigned to waitlist control groups might actively seek other online treatments since telehealth therapy is much easier to access in the postpandemic period. They might also seek in-person one-on-one therapy, which further confounds this comparison.

### Age Does Not Moderate the Intervention Effect

In terms of age, we did not find significant differences between adolescents and adults, although some previous reviews [35] argued that younger populations might be more fluent in social media and internet literacy. Therefore, they might reap larger benefits. The meta-regression results did not support this argument.

### Limitations

There are several limitations worth readers’ attention when interpreting results. First, during the moderator analysis, there are a few subgroups of moderators that have small sample sizes. For example, we only found one ES on psychological distress, 2 ESs on negative affect, and 1 study on late adulthood that met our criteria. The small sample sizes in these categories not only revealed a lack of intervention studies on these specific moderators but also reminded us to interpret these results with caution.

Moreover, the statistical power of this review was limited by the small sample size available. Since we only included rigorously designed RCTs, and only 17 studies met our inclusion criteria, our statistical power is limited, which is shown in the low degrees of freedom in the meta-regression model.

Finally, although there is a low risk of bias and the sensitivity analysis suggested that the model results were robust, the selection bias analysis suggested that the mean effect estimates were likely upwardly adjusted. Readers should be aware of this selection bias.

### Recommendations for Future Research

Based on this review, future researchers investigating social-media-based mental health interventions can focus on designing more rigorous RCTs. Researchers should focus on developing and implementing robust methodologies and exploring new social-media intervention models to effectively address mental health challenges.

We found that social-oriented programs are more effective than task-oriented programs. Future researchers can investigate the interaction between program types and other factors (ie, personality, sex, level of comfort with technology, and different mental health issues). It is possible that certain subgroups would prefer certain program orientations. Understanding this would help us design more targeted and effective programs for different users.

We found that programs with active and CAU controls are more effective than programs with waitlist control. Future studies can design 3-arm trials to assess the comparison more directly.

Last but not least, we did not collect enough information on racial distribution, which could be a valuable moderator to investigate the equity issue in the design and application of high-quality interventions. Although we planned to code for racial distribution, most studies did not report race. Even among studies that did report relevant information, they might have reported nationality instead of race, or used different ways to categorize racial groups. As most studies do not report on race, we encourage future studies to report the racial distribution of participants or consider the racial information as a variable in future RCTs of social media.

### Conclusions

Social-media-based mental health interventions provide an important modality to deliver treatment with various benefits, including accessibility, cost-effectiveness, scalability, and acceptability. Despite intense debates surrounding the effects of social media use on mental health, a more proactive approach is to explore how we can harness these platforms to design interventions that enhance mental health outcomes. As former US President Barack Obama stated [114],

*Social media is just a tool. At the end of the day, tools don’t control us. We control them, and we can remake them. It’s up to each of us to decide what we value, and then use the tools we’ve been given to advance those values*.

This meta-analysis synthesized the best evidence on this topic and found that, overall, high-quality social-media-based RCTs were effective in reducing depression, anxiety, stress, negative affect, and psychological distress. Given the benefits of scalability and cost-effectiveness of social-media-based approaches, mental health services should consider integrating online interventions into routine practice. These interventions are especially beneficial for individuals facing barriers to traditional therapy, such as those in geographically isolated areas or those unable to afford in-person care. Policymakers could consider collaborating with social media platforms to perhaps integrate these interventions into public health systems and promote equitable access to mental health care through social media platforms.

## Supplementary material

10.2196/67953 Multimedia Appendix 1 Supplementary tables.

10.2196/67953 Checklist 1 PRISMA checklist.

## Abbreviations

CAU: care as usual
ES: effect size
PRISMA: Preferred Reporting Items for Systematic reviews and Meta-Analyses
RCT: randomized controlled trial
