# Supplementary material for: Social-Media-Based Mental Health Interventions: Meta-Analysis of Randomized Controlled Trials
Source: J Med Internet Res. 2025 Aug 14;27:e67953. doi: 10.2196/67953 (PMC12352706; doi:10.2196/67953)
Supplement: Multimedia Appendix 1 [file jmir-v27-e67953-s001.docx]

**Table S1.** Search keywords.

| **Digital delivery keywords** | **Method keywords** | **Mental health keywords** |
| --- | --- | --- |
| Social media OR Facebook OR Instagram OR WhatsApp OR Twitter OR Pinterest OR LinkedIn OR Reddit OR Line OR Wechat OR Youtube OR Discord OR KakaoTalk OR Telegram OR Snapchat OR Tiktok | intervention OR program OR workshop OR module OR course OR RCT | depression OR anxiety OR stress OR ADHD OR “Anxiety sensitivity” OR Hopelessness OR “Internali* symptoms” OR “Negative affect” OR “Psychological distress” OR Self-hatred OR Trauma |

*Note.* In each database, we searched for digital delivery keywords AND method keywords AND mental health keywords as a Boolean search.

**Table S2**. Database searching details.

| **Databases** | **Round1**  **Date** | **Round1results** | **Round2**  **Date** | **Round2**  **results** | **Round 3 Date** | **Round 3 results** |
| --- | --- | --- | --- | --- | --- | --- |
| ERIC | 2005-10/17/2023 | 740 | 10/17/2023-02/24/2024 | 5 | 2024-2026 | 50 |
| PsychInfo | 2005-10/17/2023 | 1312 | 10/17/2023-02/24/2024 | 110 | 2024-2026 | 50 |
| Scopus | 2005-10/17/2023 | 1744 | 10/17/2023-02/24/2024 | 569 | 2024-2026 | 1740 |
| PsychArticles | 2005-10/17/2023 | 96 | 10/17/2023-02/24/2024 |  | 2024-2026 |  |
| Communication and Mass Media Complete | 2005-10/17/2023 | 319 | 10/17/2023-02/24/2024 | 21 | 2024-2026 | 32 |
| Proquest | 2005-10/17/2023 | 50 |  |  | 2024-2026 | 100 |
| PubMed | NA | NA | NA | NA | NA | 745 |

**Table S3**. Handsearching details.

| **Journal** | **ISSN (web)** | **Date Searched** | **Records** |
| --- | --- | --- | --- |
| Social Media + Society | 20563051 | 2005-01-01 and 2022-09-12 | 711 |
| Communication Education | 1479-5795 | 2005-01-01 and 2023-03-06 | 29 |
| Computers & Education | 0360-1315 | 2005-01-01 and 2023-03-06 | 318 |
| Computers in Human Behavior | 0747-5632 | 2005-01-01 and 2023-03-06 | 1477 |
| New media & Society | 1461-4448 | 2005-01-01 and 2023-03-06 | 535 |
| Journal of Computer-mediated communication | 1083-6101 | 2005-01-01 and 2023-03-06 | 172 |
| Cyberpsychology, Behavior, and Social Networking | 2152-2715 | 2005-01-01 and 2023-03-06 | 1876 |
| Journal of Intercultural Communication Research | 1747-5759 | 2005-01-01 and 2023-03-06 | 32 |
|  |  | Sum | 5150 |

**Table S4.** Snowballing details (Completed on 3/20/2024).

| **Citation** | **DOI** |
| --- | --- |
| The Impact of Social Media Use Interventions on Mental Well-Being: Systematic Review[1] | [10.2196/44922](https://doi.org/10.2196/44922) |
| Effectiveness of Social Media Interventions for People With Schizophrenia: A Systematic Review and Meta-Analysis.[2] | [10.2196/jmir.5385](https://doi.org/10.2196/jmir.5385) |
| Social network interventions for health behaviours and outcomes: A systematic review and meta-analysis[3] | 10.1371/journal.pmed.1002890 |
| Online and Social Networking Interventions for the Treatment of Depression in Young People: A Systematic Review[4] | 10.2196/jmir.3304 |
| Self-help Digital Interventions Targeted at Improving Psychological Well-being in Young People With Perceived or Clinically Diagnosed Reduced Well-being: Systematic Review[5] | <https://doi.org/10.2196/25716> |
| [Like this meta-analysis: Screen media and mental health.](https://psycnet.apa.org/record/2021-98715-001?doi=1)[6] | [https://doi.org/10.1037/pro0000426](https://psycnet.apa.org/doi/10.1037/pro0000426) |
| Examining the Association Between Digital Stress Components and Psychological Wellbeing: A Meta-Analysis[7] | 10.1007/s10567-023-00440-9 |
| The Use of Social Networking Sites in Mental Health Interventions for Young People: Systematic Review (Preprint)[8] | 10.2196/12244 |
| Effectiveness of Internet- and Mobile-Based Cognitive Behavioral Therapy to Reduce Suicidal Ideation and Behaviors: Protocol for a Systematic Review and Meta-Analysis of Individual Participant Data[9] | [10.3390/ijerph17145179](https://doi.org/10.3390/ijerph17145179) |
| Self-help Digital Interventions Targeted at Improving Psychological Well-being in Young People With Perceived or Clinically Diagnosed Reduced Well-being: Systematic Review[10] | 10.2196/25716 |
| Efficacy of technology-based interventions in psychosis: a systematic review and network meta-analysis[11] | [10.1017/S0033291722003610](https://doi.org/10.1017/S0033291722003610) |
| The Empowering Role of Web-Based Help Seeking on Depressive Symptoms: Systematic Review and Meta-analysis[12] | 10.2196/36964 |
| [Like this meta-analysis: Screen media and mental health.](https://psycnet.apa.org/record/2021-98715-001?doi=1)[13] | [10.1037/pro0000426](https://psycnet.apa.org/doi/10.1037/pro0000426) |
| Web-Based Interventions Supporting Adolescents and Young People With Depressive Symptoms: Systematic Review and Meta-Analysis[14] | 10.2196/mhealth.8624 |
| The effectiveness of social network interventions for psychiatric patients: A systematic review and meta-analysis[15] | [10.1016/j.cpr.2023.102321](https://doi.org/10.1016/j.cpr.2023.102321) |
| Relationship between online social support and adolescents’ mental health: A systematic review and meta-analysis[16] | [10.1002/jad.12031](https://doi.org/10.1002/jad.12031) |
| Digital technology for health promotion: Opportunities to address excess mortality in persons living with severe mental disorders[17] | 10.1136/ebmental-2018-300034 |
| Internet and Computer-Based Cognitive Behavioral Therapy for Anxiety and Depression in Youth: A Meta-Analysis of Randomized Controlled Outcome Trials[18] | [10.1371/journal.pone.0119895](https://dx.doi.org/10.1371/journal.pone.0119895) |
| Effectiveness of Social Media-based Interventions on Weight-related Behaviors and Body Weight Status: Review and Meta-analysis[19] | [10.5993/AJHB.41.6.1](https://doi.org/10.5993/ajhb.41.6.1) |
| Are Social Media Interventions for Health Behavior Change Efficacious among Populations with Health Disparities?: A Meta-Analytic Review[20] | [10.1080/10410236.2021.1937830](https://doi.org/10.1080/10410236.2021.1937830) |
| Computer therapy for the anxiety and depression disorders is effective, acceptable and practical health care: An updated meta-analysis[21] | [10.1016/j.janxdis.2018.01.001](https://dx.doi.org/10.1016/j.janxdis.2018.01.001) |
| Interventions to reduce the negative impact of online highly visual social networking site use on mental health outcomes: A scoping review[22] | [https://doi.org/10.1037/ppm0000455](https://psycnet.apa.org/doi/10.1037/ppm0000455) |
|  | references: 939  citation: 1448 |

**Table S5.** Codebook.

| **Variables** | **Explanations** |
| --- | --- |
| Drop | 1 for dropping if the study does not meet the inclusion criteria,leave it blank if it meets all the criteria |
| First Review | Name of the first reviewer |
| Second Review | Name of the second reviewer |
| Third Review | Name of the third reviewer |
| Coding finished? | Click on the checkbox if coding is finished |
| Check eligibility? | Click on the checkbox if eligibility to be included is checked |
| Authors | Name of all the authors |
| Published Year | Year when the study was published |
| Journal | Name of the journal |
| DOI/Link | DOI link of the study. |
| Study | In-text citation of the study, APAstyle, example (Yang et al., 2024) |
| Rob:6. Were outcomes assessors blind to treatment assignment? | RoB checklist item, answer using Yes, No, or Unclear |
| Rob2: Was allocation to treatment groups concealed? | RoB checklist item, answer using Yes, No, or Unclear |
| Rob4: Were participants blind to treatment assignment? | RoB checklist item, answer using Yes, No, or Unclear |
| Rob5: Were those delivering treatment blind to treatment assignment? | RoB checklist item, answer using Yes, No, or Unclear |
| Rob8: Was follow up complete and if not, were differences between groups in terms of their follow up adequately described and analyzed? | RoB checklist item, answer using Yes, No, or Unclear |
| Social media/Website | List the name of the social media platform involved in the program |
| Social media abstinence | Code as 0 or 1. 1 is when the program aims to restrain users from using social media. When it is coded as 1, the study no longer meets the eligibility criteria and should be dropped. |
| Program name | Code the name of the program. Put NA is no name is mentioned. |
| Program features | Record as reported. Can copy and paste from the main text. |
| Modality | Code as Multimedia, Text, or Video. Multimedia refers to programs that involve both text, video, and other features. |
| SocialORtask | Code as social-oriented or task-oriented. Task-oriented studies’ primary function was to assist with specific tasks. Social-oriented studies provide social interaction, emotional support, or companionship, without a specific focus on task completion. |
| CBT | Code as 1 or 0 based on whether the program used cognitive behavioral therapy. |
| Cognitive behavioral therapy? | Record as reported. |
| Targeted Outcomes | Record as reported. |
| Age | We coded this variable based on the average age of the participants in the studies. Study participants are coded to be “adolescents” if their average age is below 20, “early adulthood” if the average age ranges from 20 to below 40, “middle adulthood” if the average age ranges from 40 to below 60, and “late adulthood” if the average age is 60 and beyond. |
| Age.mean | Program participants' mean age (years) as recorded in the study |
| Age (range) | Program participants' age range (years) as recorded in the study |
| Students | Code as 0 or 1. 1 is when the program recruits student samples. |
| Country | The country(ies) the participants come from. |
| WEIRD | Acronym for Western, Educated, Industrialized, Rich, and Democratic. Code as 0 or 1 based on the reference: Beyebach M, Neipp MC, Solanes-Puchol Á, Martín-Del-Río B. Bibliometric differences between WEIRD and non-WEIRD countries in the outcome research on solution-focused brief therapy. Front Psychol. 2021;12:754885. doi:10.3389/fpsyg.2021.754885 |
| Sample size | Record as reported. |
| Female% | Record as reported. |
| Female | Record as reported. |
| Male | Record as reported. |
| White | Record as reported. |
| Black | Record as reported. |
| Hispanic | Record as reported. |
| Asian | Record as reported. |
| Other/race information | Record as reported. |
| Recruitment type | Record as reported where the participants were recruited from. |
| Special/vulnerable populations | If the program recruited general population, then code as 0. If not, report what is the special condition of the participants. |
| Clinical | Code as 1 or 0. 1 is when participants were recruited from clinical settings, such as hospitals or clinics. |
| Self.guided | Code as 0 or 1. |
| Self-guided or Supervised/Reminded by others or Guided by others | Code as guided by others or self-guided depending on the program's delivery personnel. |
| Program delivery Personnel | Code as reported who delivered the program. |
| Universal | Code as 0 or 1. |
| Targeted/Universal Intervention | Programs were coded as either universal or targeted. Universal means the program was delivered to general populations regardless of risk level. Targeted means the program was delivered to specific groups of students showing elevated levels of depression of anxiety. |
| Research Design | Code as reported. If the study is not RCT, this column flags for dropping the study. |
| Duration.weeks | Transform the duration into weeks for standardization across studies. |
| Duration | Code the duration as reported in original unites, such as minutes, hours, days, weeks, etc. |
| Number.of.sessionsORmodules | Code the number of sessions or modules as reported. |
| Single session? | If the study only has one session, we code this column as 1, which flags for exclusion. |
| Notes | This is open-text for anything worth taking note of. |
| Randomized | Code as 0 or 1. If the participants were not randomly allocated to treatment or control groups, this flags for exclusion. |
| Clustered | Code as 0 or 1 based on whether the study design is cluster randomization. |
| Treatment.N.original | Number of treatment participants at the baseline assessment |
| Control.N.original | Number of control participants at the baseline assessment |
| Treatment.N.post-test | Number of treatment participants at the post-test assessment |
| Control.N post-test | Number of control participants at the post-test assessment |
| Attrition | This column has imbedded equation to compute from previous columns the attrition percentage from pre-test to post-test. |
| Differential Attrition | This column has imbedded equation to compute from previous columns the differential attrition percentage between treatment and control groups. |
| Treatment.Cluster | Only code this column is the study is cluster RCT. Code the number of treatment clusters. |
| Control.Cluster | Only code this column is the study is cluster RCT. Code the number of control clusters. |
| Unit (schools, classes, couple) | Only code this column is the study is cluster RCT. Code the unit of clusters. |
| Control.Waitlist | Code as 0 or 1 depending on whether the control group used waitlist control design. |
| Control group | Code as reported the control group design. |
| Negative Outcomes | Code as 0 or 1. 1 when the outcome is a negative mental health issue, such as depression, anxiety, stress, etc. |
| Positive Outcomes | Code as 0 or 1. 1 when the outcome is a positive mental health outcome, such as well-being, life satisfaction, etc. If this column is coded as 1, it flags for exclusion. |
| Outcomes | Code as reported the outcome measured. |
| Measure | Code the scale used to measure the outcome |
| # of Items | Code the number of items used in this scale |
| Citation | Code the reference for this scale using APA formatting |
| Cronbach's alpha | Code as reported the Cronbach's alpha for this scale. |
| MeasureDescription | Code as reported the descriptio of this measurement |
| Baseline differences_T, <0.25 | This column automatically computes the baseline difference between the treatment groups based on other columns coded. |
| Baseline differences_C, <0.25 | This column automatically computes the baseline difference between the control groups based on other columns coded. |
| T_Mean_Pre | Pre-test treatment mean value |
| T_SD_Pre | Pre-test treatment standard deviation value |
| C_Mean_Pre | Pre-test control mean value |
| C_SD_Pre | Pre-test control standard deviation value |
| T_Mean_Post | Post-test treatment mean value |
| T_SD_Post | Post-test treatment standard deviation value |
| C_Mean_Post | Post-test control mean value |
| C_SD_Post | Post-test control standard deviation value |
| ES | Effect size as reported |
| Follow-up | Code as 0 or 1. 1 is when there is a follow-up from the post-test. |
| Duration (XX-month follow up) | Code when is the follow-up, as in how many months after the post-test. |
| Contact author? Corresponding author's name and email | Code the name and email of the corresponding author in case we need to reach out for missing data or questions. |

**REFERENCES**

1. Plackett R, Blyth A, Schartau P. The impact of social media use interventions on mental well‑being: systematic review. J Med Internet Res. 2023;25:e44922. doi:10.2196/44922
2. Välimäki M, Athanasopoulou C, Lahti M, Adams CE. Effectiveness of social media interventions for people with schizophrenia: a systematic review and meta‑analysis. J Med Internet Res. 2016 Apr 22;18(4):e92. doi:10.2196/jmir.5385
3. Hunter RF, de la Haye K, Murray JM, Badham J, Valente TW, Clarke M, et al. Social network interventions for health behaviours and outcomes: a systematic review and meta‑analysis. PLoS Med. 2019 Sep 3;16(9):e1002890
4. Rice SM, Goodall J, Hetrick SE, Parker AG, Gilbertson T, Amminger GP, Davey CG, McGorry PD, Gleeson J, Alvarez‑Jiménez M. Online and social networking interventions for the treatment of depression in young people: a systematic review. J Med Internet Res. 2014 Sep 16;16(9):e206. doi:10.2196/jmir.3304
5. Babbage CM, Jackson GM, Davies EB, Nixon E. Self‑help digital interventions targeted at improving psychological well‑being in young people with perceived or clinically diagnosed reduced well‑being: systematic review. JMIR Ment Health. 2022 Aug 26;9(8):e25716. doi:10.2196/25716
6. Ferguson CJ, Kaye LK, Branley‑Bell D, Markey P, Ivory JD, Klisanin D, et al. Like this meta‑analysis: screen media and mental health. Prof Psychol Res Pract. 2022 Apr 1;53(2):205–214. doi:10.1037/pro0000426
7. Khetawat D, Steele RG. Examining the association between digital stress components and psychological wellbeing: a meta‑analysis. Clin Child Fam Psychol Rev. 2023 Jul;26(4):957–74. doi:10.1007/s10567-023-00440-9
8. Ridout B, Campbell A. The use of social networking sites in mental health interventions for young people: systematic review (Preprint). J Med Internet Res. 2018 Dec 18;20(12):e12244. doi:10.2196/12244
9. Büscher R, Beisemann M, Doebler P, Steubl L, Domhardt M, Cuijpers P, et al. Effectiveness of internet‑ and mobile‑based cognitive behavioral therapy to reduce suicidal ideation and behaviors: protocol for a systematic review and meta‑analysis of individual participant data. Int J Environ Res Public Health. 2020 Jul 2;17(14):5179. doi:10.3390/ijerph17145179
10. Babbage CM, Jackson GM, Davies EB, Nixon E. Self‑help digital interventions targeted at improving psychological well‑being in young people with perceived or clinically diagnosed reduced well‑being: systematic review. JMIR Ment Health. 2022 Aug 26;9(8):e25716. doi:10.2196/25716
11. Morales‑Pillado C, Fernández‑Castilla B, Sánchez‑Gutiérrez T, González‑Fraile E, Barbeito S, Calvo A. Efficacy of technology‑based interventions in psychosis: a systematic review and network meta‑analysis. Psychol Med. 2023;53(13):6304–15. doi:10.1017/S0033291722003610
12. Bizzotto N, Marciano L, de Bruijn GJ, Schulz PJ. The empowering role of web‑based help seeking on depressive symptoms: systematic review and meta‑analysis. J Med Internet Res. 2023 Feb 2;25:e36964. doi:10.2196/36964
13. Ferguson CJ, Kaye LK, Branley‑Bell D, Markey P, Ivory JD, Klisanin D, et al. Like this meta‑analysis: screen media and mental health. Prof Psychol Res Pract. 2022 Apr 1;53(2):205–14. doi:10.1037/pro0000426
14. Välimäki M, Anttila K, Anttila M, Lahti M. Web‑based interventions supporting adolescents and young people with depressive symptoms: systematic review and meta‑analysis. JMIR Mhealth Uhealth. 2017 Dec 8;5(12):e180. doi:10.2196/mhealth.8624
15. Swinkels LTA, Hoeve M, ter Harmsel JF, Schoonmade LJ, Dekker JJM, Popma A, van der Pol TM. The effectiveness of social network interventions for psychiatric patients: a systematic review and meta‑analysis. Clin Psychol Rev. 2023 Aug;104:102321. doi:10.1016/j.cpr.2023.102321
16. Zhou Z, Cheng Q. Relationship between online social support and adolescents’ mental health: a systematic review and meta-analysis. J Adolesc. 2022 Apr;94:281–92. doi:10.1002/jad.12031
17. Naslund JA, Aschbrenner KA, Bartels SJ. Digital technology for health promotion: opportunities to address excess mortality in persons living with severe mental disorders. Evid Based Ment Health. 2018 Feb;21(1):17–22. doi:10.1136/ebmental-2018-300034
18. Ebert DD, Zarski A‑C, Christensen H, Stikkelbroek Y, Cuijpers P, Berking M, Riper H. Internet and computer-based cognitive behavioral therapy for anxiety and depression in youth: a meta-analysis of randomized controlled outcome trials. PLoS One. 2015 Mar 18;10(3):e0119895. doi:10.1371/journal.pone.0119895
19. An R, Ji M, Zhang S. Effectiveness of social media-based interventions on weight-related behaviors and body weight status: review and meta-analysis. Am J Health Behav. 2017;41(6):670–682. doi:10.5993/AJHB.41.6.1
20. Bender JL, Choi GJ, Ahn JH, Fukuoka Y, Kim K. Are social media interventions for health behavior change efficacious among populations with health disparities? A meta-analytic review. Health Commun. 2021;36(5):613–624. doi:10.1080/10410236.2021.1937830
21. Andrews G, Basu A, Cuijpers P, Craske MG, McEvoy P, English CL, Newby JM. Computer therapy for the anxiety and depression disorders is effective, acceptable and practical health care: an updated meta-analysis. J Anxiety Disord. 2018;55:70–78. doi:10.1016/j.janxdis.2018.01.001
22. Herriman Z, Taylor AM, Roberts RM. Interventions to reduce the negative impact of online highly visual social networking site use on mental health outcomes: a scoping review. Psychol Pop Media. 2023;13(1):111–139. doi:10.1037/ppm0000455
